# What is a healthy dating relationship and what helps it get there? Perceptions of Chilean adolescents from a gender and inclusivity perspective

**DOI:** 10.3389/fpsyg.2025.1489208

**Published:** 2025-07-24

**Authors:** Christianne Zulic-Agramunt, Ana Poo-Figueroa, Gabriel Gatica-Bahamonde, Nicolás Salazar, Andrea Saldana, Bastian Carter-Thuillier, Carles Pérez-Testor

**Affiliations:** ^1^Department of Mental Health and Psychiatry, University of La Frontera, Temuco, Chile; ^2^Department of Psychology, Ramón Llull University, Barcelona, Spain; ^3^Department of Psychology, University of La Frontera, Temuco, Chile; ^4^Sección de Psiquiatría del Niño y del Adolescente, División de Neurociencias, Facultad de Medicina, Pontificia Universidad Católica de Chile, Santiago, Chile; ^5^Department of International Health, Institute for Public Health and Care Research, Faculty of Health, Medicine and Life Sciences, Maastricht University, Maastricht, Netherlands; ^6^Department of Education, Universidad de Los Lagos, Osorno, Chile; ^7^Faculty of Education, Catholic University of Temuco, Temuco, Chile; ^8^Faculty of Education and Social Sciences, Andrés Bello University, Santiago, Chile

**Keywords:** healthy relationships, dating violence, adolescents, protective factors, risk factors

## Abstract

**Introduction:**

Relationships are often the focal point of life and can have a positive or a negative impact on a child's or adolescent's development, thus understanding the traits of healthy dating relationships may benefit wellbeing during this period of life and into adulthood.

**Methods:**

In this transversal study, a sample of 65 adolescents between 10 and 19 years of age were recruited from schools in Araucanía and metropolitan regions of Chile during 2023, and they were distributed into 10 focus groups stratified by gender and age. A qualitative methodology with a phenomenological approach was used. Content análisis was performed stratified by age group (10–14 years, 15–19 years) and gender identity.

**Results:**

Information was obtained from adolescents about what they considered a healthy relationship, making distinctions between different types of unhealthy relationships and capturing subtleties about what they considered protective and risk factors. The adolescents were not more consumers of information; they required commitment and support in their development processes.

**Discussion:**

Listening to their own voice could help generate solutions that made more sense to them. In the view of an adolescent, the formation of romantic couples was a fundamental and important issue that should be considered in public policies that promote health and prevent dating violence and its consequences.

## 1 Introduction

Some authors have defined a healthy romantic relationship as one characterized by strong communication and negotiation skills, caring behavior, self-expression, respect, trust, honesty, and fairness. These characteristics are considered necessary along with the absence of abuse in the relationship (Gómez-López et al., 2019; Hielscher et al., 2021).

From a developmental perspective, adolescence, the period between ages 10 and 19 years (Gómez-López et al., 2019), and emerging adulthood (the periods spanning the second and third decades of life) have been described as vitally important to romantic relationships (Connolly and McIsaac, 2009; Arnett et al., 2014; Arnett, 2000; Collins, 2003; Gómez-López et al., 2019). Defined as “mutually acknowledged, ongoing voluntary interactions”, these relationships, unlike others such as friendships, are characterized by a particular intensity, specific expressions of affection, and the initiation of erotic sexual encounters (Collins, 2003; Collins et al., 2009).

These experiences are frequent during these stages of development and tend to consolidate over time, representing an important context of learning and training for future intimate relationships. By mid-adolescence, most children have been involved in at least one romantic relationship, providing them with an environment characterized by increased intimacy, support, and importance as they grow older. According to developmental task theory, during adolescence, romantic involvement is an emerging developmental task, which will eventually become a salient developmental task in adulthood (Connolly et al., 2014; Carver et al., 2003; Connolly and McIsaac, 2009; Shulman et al., 2011; Furman and Collibee, 2014; Gómez-López et al., 2019).

From this perspective, healthy romantic relationships, when sustained over time, can even achieve a transformation of the attachment bond, recognizing that the quality of the relationship, the history of shared experiences, the sense of attachment, and the beliefs that emerge from the entire experience, manage to modulate the wellbeing of the couple (Casad et al., 2014; Zimmer-Gembeck and Ducat, 2010). These characteristics are considered necessary in addition to the absence of abuse in the relationship (Hielscher et al., 2021).

Although romantic relationships during adolescence are common and vitally important (e.g., in the United States, almost 75% of 13- to 16-year-olds reported they were dating or had dated someone), much of the evidence collected pertains to adolescents older than 15 years (Muñoz-Rivas et al., 2022); despite the fact that today, dating relationships begin at increasingly earlier ages (Organización Mundial de la Salud, 2024), there is a lack of knowledge about how this age group relates or even what their ideas about what it means to build a relationship are, and this lack of knowledge makes it difficult to understand in a deeper and more complex way how these relationships are configured, as well as their true impact on the different areas and stages of human development (Hossain et al., 2020; Gómez-López et al., 2019).

To help young people understand what it means to have a healthy relationship, most intervention programs recommend that the program begins before teens begin dating, further justifying the importance of conducting studies at earlier ages. They also emphasize the importance of creating dialogues and spaces in which knowledge can be provided as a basis for instilling beliefs and attitudes that support healthy relationships and reject violence, since both awareness and tools are needed to promote healthy relationships and avoid the traumatic and life-changing experience of domestic violence (DV; Sanhueza et al., 2022; Schubert, 2015).

The desire for interpersonal attachment, that is, the need to belong, has been described as a fundamental human motivation and is essential for wellbeing, especially when it refers to romantic relationships. These have been identified as an important source of emotional bonding and as an element that can contribute to the development of a positive self-concept and better social integration and thus, have important repercussions in later stages of life, favoring the physical and mental health of people (Baumeister and Leary, 1995; Reis et al., 2000; Gómez-López et al., 2019). This was confirmed in the study by Diener and Seligman (2002), which showed that extremely happy people establish good and solid interpersonal relationships, which invites the participants to reflect on wellbeing, even at a relational level, as something more than the absence of illness, where it is necessary to establish frames of reference to understand what constitutes a healthy relationship [World Health Organization (WHO), 2014; Cooke et al., 2016; Seligman and Csikszentmihalyi, 2000; Keyes, 2002; Keyes and Waterman, 2003; Gómez-López et al., 2019].

Unfortunately, psychology has focused on repairing damage within a disease model of human functioning, paying almost exclusive attention to pathology and neglecting the study of the positive characteristics that make life worth living. It is currently known that the absence of pathology does not necessarily correlate with positive dimensions of health and wellbeing, which has generated gaps regarding the terminological diversity found in the different studies, including relational ones, giving rise to a certain degree of controversy in the different topics related to one's own health. Although this situation has contributed to a productive scientific debate, it has also generated considerable theoretical and methodological ambiguity and confusion, which is also reflected in the different terminologies in this regard in a broad way, including those used for relationships (Hielscher et al., 2021; Keyes, 2002; Keyes and Waterman, 2003; Vázquez, 2006). And while there is broad consensus in the literature that love is one of the strengths most closely linked to personal wellbeing and happiness and that it is associated with higher rates of self-esteem, security, life satisfaction, positive affect, and achievement of personal and relational goals, little research has focused on studying how adolescents relate romantically, and even fewer have focused on healthy ways of relating (Blanca et al., 2017; Park et al., 2004; Davila et al., 2017; Dush and Amato, 2005; Gómez-López et al., 2019).

### 1.1 Support from the ecological model

The ecological model (López and Guiamaro, 2017) has helped us understand the basis of the factors that support the development of healthy and unhealthy relationships. This model, unlike Bronfenbrenner's, emphasizes the interaction between the child and their environment, considering the family as a fundamental area for their development, underscoring the need for a collaborative relationship between the family and the school for the child to achieve optimal development. Regarding the factors that promote healthy relationships, from this comprehensive model, the following stood out: high self-esteem at the individual level, positive relationships between parents and children, feeling supported by parents and caregivers who provide a good relationship model at the family level (Dardis et al., 2014; Hébert et al., 2017; Malhotra et al., 2014; Spencer et al., 2019; Taylor et al., 2017; Vagi et al., 2013), prosocial peer networks, a neighborhood under control and with healthy relationships, good attachment to school, and active participation both in school and in the neighborhood at the structural level (Johnson et al., 2015; Taquette and Monteiro, 2019; Taylor et al., 2017; Vagi et al., 2013). In terms of the structural level, teachers and school staff have been highlighted as the main agents of change and the people responsible for education related to gender values (Pérez-Marco et al., 2020). It is assumed that the motivation to adopt health-promoting behaviors, including at the relational level, is a function of personal values and social context (Brar et al., 2022), and that it is constructed on the basis of information received from the different settings inhabited by a person. Under this same model, romantic relationships have also been associated with negative outcomes, especially during adolescence, and with risk factors that, on the contrary, have suggested that romantic involvement may be related to the presence of different forms of violence, which has been associated with externalizing and internalizing symptoms in those who suffer from them, such as depression, anxiety, poor academic performance, low self-esteem, substance and alcohol use, eating disorders, post-traumatic stress disorder, personality disorder, worse psychosocial functioning or delinquency, self-harm and even suicidal behavior (Ackard and Neumark-Sztainer, 2002; Boyle and O'Sullivan, 2013; Cui et al., 2011; Miller, 2014; Peñúñuri et al., 2019; Soller, 2014; Taquette and Monteiro, 2019; Viejo, 2014; Zimmer-Gembeck et al., 2001). Thus, we note that romantic relationships are often at the center of life and provide positive or negative experiences that can influence short- and, more importantly, long-term development, impacting interpersonal relationships and the wellbeing of individuals in systemic and transgenerational ways (Gila-Ordóñez and Callejón-Chinchilla, 2018; Gómez-López et al., 2019).

### 1.2 Sociocultural context

Accounting for the importance of psychosocial determinants, the characteristics of the location where this study was carried out must be considered. In Chile, patriarchy is prominent (Calvin et al., 2013) due to the influence of Spanish colonization and the Catholic Church. In Chilean culture, rigid gender roles, the acceptance of interpersonal violence as a solution to conflicts, and sexist and machismo values stand out, and there is an indigenous population of 1,565,915 individuals, that is, 9% of the national population. Of these, the Mapuche people represent 84%, concentrated mostly in the Araucanía and metropolitan regions, and they are characterized by a high prevalence of gender and interpersonal violence. In this regard, the ancestral roles of men and women have been modified as a result of cultural mixing, integrating the patriarchal concepts of the Western family into their identity (Calfío, 2009).

For the present study, it is also important to bear in mind that although Spanish is spoken in Chile, the culture includes elements not only of Spanish culture but of Mapuche culture, as well, reflected in the fact that the word “pololear” is commonly used to describe dating. This word comes from the Mapuche language (Mapudungun, or “language of the earth”) and originates from the word “puldu”, which means beetle or blowfly (Unidad de Coordinación de Asuntos Indígenas, 2021). Thus, in Chile, just as a fly hovers around ripe fruit, a “pololo” is a man who hovers around a woman with the aim of attracting her.

Regarding Chilean legislation, recently, on November 23, 2021, Law No. 21,393 was officially published, which designates 7 February as the “National Day of Non-violence in Pololeo” (Chile, 2021), but there is still no law that sanctions this problem. The current Law No. 20,066 (Chile, 2005) sanctions DV that occurs exclusively between people united by marriage, consanguinity, or kinship (e.g., former couples who have a child in common), affinity, or conjugal coexistence, but not DV, making its seriousness invisible, which especially affects young people and adolescents (Valdivia-Peralta et al., 2018).

### 1.3 Gender and inclusion

Consistent with the Pan American Health Organization (Organización Mundial de la Salud and Organización Panamericana de la Salud, 2013), which has emphasized that, from a gender equity perspective, it is necessary to prevent DV early in order to modify the stereotypes and cultural factors that support it from childhood and adolescence and perpetuate it in adulthood as a stable model of relational behavior (Calvin et al., 2013; Guevara et al., 2017; Organización Mundial de la Salud and Organización Panamericana de la Salud, 2013; Sanhueza et al., 2022; Zamudio, 2012), this study included the gender variable among those studied.

Although the gender variable has been established in the dynamics of DV, little research has considered gender as a variable *per se* in its samples (i.e., considering only the sex of the participants), which limits the conclusions regarding the role of gender in the dynamics of DV. This has also led to a lower level of inquiry into gender diversity, because sex generally implies a binary thinking model and often leads to an underrepresentation of the population that shows gender diversity, where it is relevant to ask about the role of the psychosocial stress observed in minorities and how this can affect the ways of relating as a couple (Espelage et al., 2016; Otero et al., 2015).

### 1.4 Purpose of this study

Consistent with the above and considering the gap in the research on what constitutes a healthy romantic relationship, because the few studies that have focused on healthy relationships tend to frame them in terms of the absence of unhealthy dynamics, and most have focused mainly on dating violence (e.g., DV), this study aims to answer the following research question: What do Chilean adolescents understand about a healthy relationship, and what factors do they consider important for fostering it, from their perspective? So, this study breaks with the existing research paradigm and considers belonging to a culture with certain peculiarities to identify the meaning of a healthy relationships. Moreover, it endeavors to describe, what factors were considered relevant in promoting them. Also, we queried their distinctions that, from their point of view, must be taken into account to help them from falling into unhealthy or violent relationships, from a gender and inclusive perspective.

## 2 Materials and methods

### 2.1 Design

This was a qualitative cross-sectional study with a phenomenological, descriptive approach (Fuster, 2019; Hernández-Sampieri and Mendoza, 2018). Phenomenology is interested in making a detailed description of the experience lived by the participants, revealing their meanings (Castillo, 2021).

### 2.2 Participants

For this study, the sample inclusion criteria were: Chilean adolescents aged between 10 and 19 years; adolescents attending municipal schools, charter schools, or high schools from different parts of the central-southern region of the country; and adolescents who had mastered reading and writing. Adolescents who, due to cognitive difficulties, were unable to complete a self-report questionnaire or understand instructions or informed assent were excluded.

Using the intentional convenience sampling technique, accessing the adolescent council of the Ministry of Health of Chile and through contacts granted by the respective superintendencies of education, the formation of focus groups was convened to respond to a matrix pre-defined in our research design.

Prior to the above, a survey was carried out to collect socio-demographic data. The survey was answered by 92 adolescents with an average age of 16 years, of whom 24% belonged to the third grade (secondary), 16% to the fourth grade (secondary), 20% to the eighth grade (primary), and 12% to the sixth grade (primary). Regarding their places of origin, 40% were from the Araucanía region, 15% from the metropolitan region, 3% from the Maule region, and 2 and 42% were from another region of Chile. Regarding sex, 56.5% were female, 39% were male, and 1% were intersex.

Regarding gender, 47% had a female gender identity, 39% had a male gender identity, 6% were transexual, and 4% had a fluid gender identity. With respect to sexual orientation, 71% identified as heterosexual, 14% indicated they did not feel sure about it, 10% were bisexual, and 2% were homosexual. Regarding ethnicity, 77% said they did not belong to an indigenous people and 23% said they belonged to the Mapuche indigenous people, while 95.7% reported being of Chilean nationality. Per dating, 62% had had a dating relationship and 44% were in a dating relationship, while 12% reported having felt abused in a dating relationship, and 71% replied that the gender corresponding to the relationship in which they felt abused was male (Table 1).

**Table 1:** Sociodemographic data.

**Category**	**Subcategory**	**Frequency**	**Cumulative frequency**	**Percentage**
Sex	Men	39	0.42	42%
	Women	52	0.57	57%
	Intersex	1	0.01	1%
Gender	Female	47	0.51	51%
	Masculine	36	0.39	39%
	Trans	5	0.06	6%
	Fluid	4	0.04	4%
Sexual orientation	Heterosexual	65	0.71	71%
	Homosexual	2	0.02	2%
	Bisexual	9	0.10	10%
	I'm not sure	13	0.14	14%
	Other	3	0.03	3%
Age	10–15 years	48	0.52	52%
	16–20 years	44	0.48	48%
Scholarship	5th grade (primary)	6	0.07	7%
	6th grade (primary)	11	0.12	12%
	7th grade (primary)	5	0.05	5%
	8th grade (primary)	18	0.20	20%
	1st grade (secondary)	10	0.11	11%
	2nd grade (secondary)	5	0.05	5%
	3rd grade (secondary)	22	0.24	24%
	4th grade (secondary)	15	0.16	16%
Place of origin	Araucania region	36	0.40	40%
	Metropolitan region	14	0.15	15%
	Maule region	3	0.03	3%
	Other	39	0.42	42%
Belonging to an indigenous people	Yes	21	0.23	23%
	No	71	0.77	77%
Nationality	Chilean	88	0.96	96%
	Venezuelan	1	0.01	1%
	Other	3	0.03	3%
Have you been in a dating relationship?	Yes	57	0.62	62%
	No	35	0.38	38%
	**Total**		**92**	**1.00**	**100%**
Are you currently in a relationship?	Yes	25	0.44	44%
	No	32	0.56	56%
Have you felt mistreatment in a relationship?	Yes	7	0.12	12%
	No	50	0.88	88%
**Total**		**57**	**1.00**	**100%**
What was the gender of your partner in the relationship in which you felt mistreated?	Female	2	0.29	29%
	Male	5	0.71	71%
Other	0	0	0
**Total**		**7**	**1.00**	**100%**

Our own elaboration.

Of the 92 adolescents, 65 attended one of our focus groups, but the remaining 27 did not respond to the call. Participants were allocated into groups according to a two-way matrix, considering age and gender. Two age ranges were defined: 10–14 years and 15–19 years, and gender: female, male, and diverse. Thus, a final sample of 65 adolescents was obtained, distributed into 10 focus groups stratified by gender and age. Adolescents who identified with a gender other than their birth sex were grouped into groups called “diverse groups” (DG; Table 2).

**Table 2:** Description of data recollection.

**Code**	**Gender identity**	**Age**	**Participants**	**Mode**
H10-14 P	Men	10–14	7	In person
H10-14V	Men	10–14	6	Virtual
M10-14P	Women	10–14	13	In person
M10-14V	Women	10–14	5	Virtual
H15-19P	Men	15–19	6	In person
H15-19V	Men	15–19	6	Virtual
M15-19P	Women	15–19	9	In person
M15-19V	Women	15–19	4	Virtual
DG10-14P	X (2 TF and 2 TM)	10–14	4	In person
DG15-19P	X (2 TF and 3 TM)	15–19	5	Virtual
TOTAL			65	

Stratification.

Our own elaboration.

### 2.3 Procedure

A broad call was made in light of the inclusion criteria to recruit the study's sample through ministerial cadastres and key contacts through social networks. Initially, government institutions, education regional ministerial secretaries (SEREMIS) and health services of the Araucanía and metropolitan regions were contacted.

Access to the adolescent advisory council of the Ministry of Health (MINSAL) was requested through a meeting held through the Chilean Lobby Law. In all institutions that agreed to participate, a motivational video aimed at parents and adolescents was broadcast.

To obtain the participation of adolescents under 18 years of age, parents were sent an in-formed consent form that explained to them that their child's participation would be voluntary, that the discussions would be recorded through audio recordings and field notes, and that the participants could withdraw from the research at any time and access the results if they wished. Likewise, the confidentiality of the data was emphasized by the researchers, as well as the fact that the data would be used for research purposes only. Once the adolescent's consent form was signed, the platform directed them to a survey that assessed socio-demographic data, the person's gender, and their history of previous relationships.

As for the focus groups, five were held virtually and five in person over 6 months, and each lasted ~90 min. These were conducted by two researchers who were child and adolescent psychiatrists or psychologists. The data collection technique used was a focus group, organized according to a script (Tümen and Ahmed, 2021), with open questions developed by the authors and triangulated by experts. For the focus groups, five were held virtually and five in person over 6 months, and each lasted ~90 min. These were conducted by two researchers who were child and adolescent psychiatrists or psychologists. The focus groups varied in length. The median number of participants in the focus groups was 6. One group had 13 participants, who were accepted given the adolescents' motivation to participate. In this group, the time was longer, allowing all participants to express their opinions. The script began with each participant saying the first thing that came to mind when the word “couple” was mentioned.

The script began with each participant saying the first thing that came to mind when the word “couple” was mentioned. Participants were then asked to comment on what a healthy relationship was for them compared to an unhealthy one. They were then asked to comment on: (a) Why are there healthy and unhealthy relationships? (b) What is important to have for healthy and unhealthy relationships? (c) What causes violence in relationships? (d) What would they recommend to reduce violence in relationships? (e) How can one best detect and address it? and (f) Would they like to ask the group another question on the topic in question?

Likewise, the confidentiality of the data were emphasized by the researchers, as well as the fact that they would be used only for research purposes. The study was part of the DIUFRO INI DI21-0100 Project, which was approved by the Ethics Committee of the University of La Frontera and Southern Araucanía Health Service, Temuco, Chile.

In the event of screening an adolescent at risk, referral to the polyclinic of the same university and counseling for parents and educational networks to activate the corresponding health referral network were proposed. Furthermore, at the end of the research, training on the topic was provided for the schools.

### 2.4 Analysis plans

**Figure 1:**
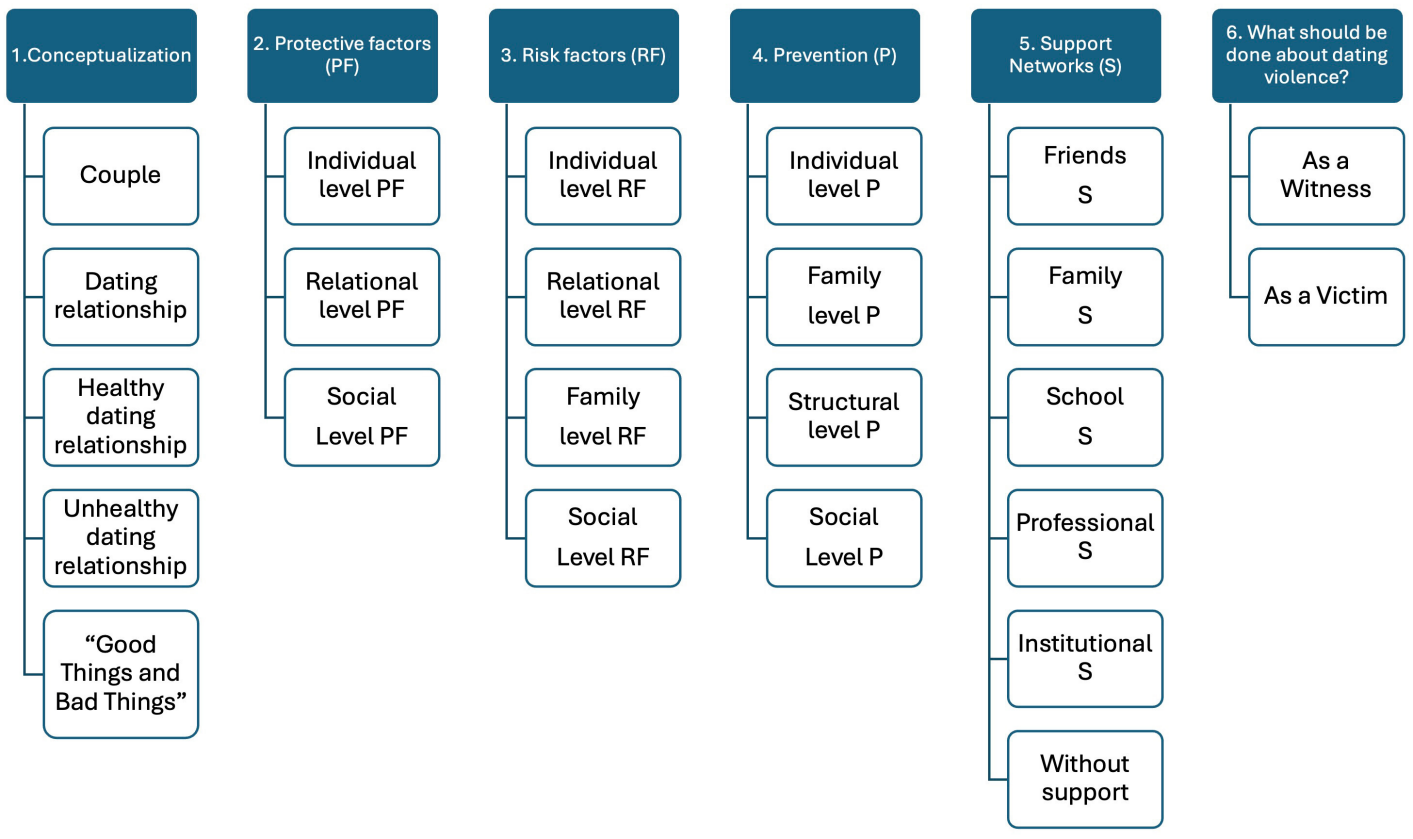
Red lines indicating the Atlas.ti (2022) software-derived cores and categories. From the qualitative analysis, six cores emerged that were subdivided into different categories that allowed us to explore and describe the different, most relevant aspects of what a healthy dating relationship entails, as indicated by the participating adolescents.

The information obtained was transcribed and the participants were identified with a code to protect confidentiality. Content analysis was then conducted through a first level of open coding, transforming the units through an inductive process into categories, to which a concept representing their meaning was assigned. The second level of coding consisted of comparing the categories to identify similarities, differences, and possible links between them. In this process, the categories were reduced until the central elements of the analysis were reached (Hernández-Sampieri and Mendoza, 2018). The central themes emerged directly in relation to the questions posed in the script, which was developed from the literature review (Brar et al., 2022; Collins, 2003; Collins et al., 2009; Connolly et al., 2014; Davila et al., 2017). These were called core elements: Conceptualization, Protective Factors, Risk Factors, Prevention, Support Networks, and What Should be Done About Dating Violence. Each of these was then explored in greater depth, based on what emerged from the discussion in each focus group. The content analysis through open and axial coding was supported with ATLAS.ti 2022 software (Figure 1). Validation of the results was ensured by saturation of the information (the point at which collecting new data no longer generates new insights or relevant themes for the study. It is reached when the data no longer replicate information) and triangulation by the researcher in the process of data coding and analysis (three researchers with different perspectives are employed to analyze the data, which can reduce bias and enrich interpretation), and by means of verifiability through external review.

## 3 Results

Upon saturation of the responses, for the topics associated with a healthy dating relationship (HDR), the following core elements emerged: Conceptualization, Protective Factors, Risk Factors, Prevention, Support Networks, and What Should be Done About Dating Violence. The following results display the core elements in bold with initial capital letters, the categories in bold italics with initial capital letters, and the subcategories that emerged from analyzing the information provided by the participants in the focus groups in lower case italics (see Figure 1). For a better understanding, most of the descriptions include quotations that exemplify the above.

### 3.1 Conceptualization of a dating relationship

Concerning the first core element, **Conceptualization** (186 quotes), the participants stated that a **Couple** relationship has attraction, intimacy, companionship, and mutual support and does not necessarily imply a healthy relationship in every case, although should, according to them. They also stated that a couple relationship can occur between people of different genders. Some believed that a member of a couple does not necessarily supply the other's unmet needs, and that being a couple does not necessarily imply seeking complementarity. In contrast, others thought that being part of a couple is the only way to achieve wholeness and completeness. This was exemplified in the following quotations: “A companion, someone who supports you and accompanies you, is not another half or a better half, it's someone who supports you and you accompany each other” (Orlando. M 15-19, V); also reported: “A partner can be someone who is not simply like a complete whole. I think we are all incomplete to the point of getting to know a person for whom we do things we didn't know we could do” (Andrés. H 15-19, Z).

As for a dating relationship, they described it as a deep relationship between two people, where the most relevant things are attraction and love, and which implies a short time of being together: “Dating is when two people fall in love and maintain a relationship” (Juana. F 10-14, I). They considered that a longer relationship (years) would indicate a couple relationship.

An HDR was described as a “process” rather than an event, based on trust, respect for space, time, individuality, mutual support, and commitment to each other. Affection, love, and care for the other are present and also imply that each has personal development and independence, promoting self-knowledge. A participant shared: “I think trust is the main thing, also mutual support and feeling that one is meant for the other always, at all times, that one is not going to abandon the other overnight” (Juan. M 15-19, V); “It's more important to have each other with respect to what is self-perception. You see? Like they support you in being yourself. It doesn't matter how much time you spend or the moments you spend, but that's what's important, I think” (Anita. F 10-14, I).

An **Unhealthy Dating Relationship** was described as a relationship where there is no connection, little communication, no respect for boundaries, no mutual support, even egocentrism, deceit, control, fights, jealousy, emotional dependence, use of the other to satisfy one's own needs, humiliation, and violence, which was expressed in the following ways: “Some people almost always talk about themselves, and that goes hand-in-hand with the lack of communication. ‘You know I feel like this…' and we don't listen to what the other person says” (Fernando. M 15-19, V).

Some adolescents were aware that a relationship could not be categorically defined as “good” or “bad” since, in most relationships, according to the participants, there were **Good Things and Bad Things**. It was to be expected that there would be conflicts, differences of opinion, and arguments, and that the important thing was to resolve disputes in a non-violent way, to know how to apologize, reach agreement, and reconcile, as reflected in the following quotation: “I think it's difficult, because, at the end of the day, no couple is perfect, for example, to say ‘I've never done this', ‘we've never had a fight' or ‘we've never had arguments', is somewhat unlikely” (Cristian. M 15-19, V).

### 3.2 The protective factors of HDRs

Regarding the second core element of **Protective Factors** (nine quotations) that would foster an HDR, consistent with the ecological model, the first thing that stood out was that the teenagers literally used the words “protective factor” to describe those things that would support a healthy relationship. Regarding those, the adolescents pointed out that at the **Individual Level**, emotional independence and self-esteem were important; for example, they stated: “I find that to love somebody you first have to love yourself” (Jessica. F 10-14, V).

At the **Relational Level**, they expressed that it was necessary to establish a relationship in which one must take care of the other and not harm the other, in order to generate trust. Participants pointed out that this implied affective responsibility, as exemplified in the following quotation: “You feel responsible for the other” (Marcela. F 10-14, I).

At the **Social Level**, they mentioned the fact that society included this topic in the conversation and that, as a result, they were much better informed. They also recognized a cultural shift in attitude that encouraged respect and rejected violence, which was expressed in the following quotation: “Times have changed a lot from what people used to think thanks to information, and technology has made many important cases visible about this topic” (José. M 15-19, I).

### 3.3 The risk factors that hinder HDRs

The participants made extensive reference to the third central element of our analysis, literally using the words “risk factors” (215 quotes, in total) to describe those factors that they identified that would make it difficult to achieve a healthy relationship.

At the **Individual Level**, the adolescents referred to the personal characteristics of individuals exposed to DV, highlighting the immaturity inherent to adolescence, insecurity, low self-esteem, and mental health issues like substance abuse and depression, as well as idealization and the search for romantic love. Others recognized that anyone could be exposed to dating violence, de-stigmatizing partners who suffer violence, which is expressed in the following quotation: “I think it refers to someone idealizing the person when they're ‘in love' and it's like you don't find anything wrong with the person, like you suddenly justify the bad attitudes, like you lose the ability to criticize your partner … because everything they do is okay” (Camila. F 15-19, I).

Participants pointed out that there were people “crazy in the head”, who define themselves as abnormal in terms of managing their impulsiveness and aggressiveness, and who have a low capacity for emotion regulation, causing harm intentionally or unconsciously. The latter was explained by normalization of violence from the context, exemplified in this statement: “I don't think they sit down one day to plan what they are going to do. No, I think they're just like that, and they have a problem in their head, they're crazy, and they're just like that” (Andrea. F 15-19, V).

Another aspect that stood out was emotional dependence; in general, the participants described this category essentially as the vulnerability experienced by a person who needs to feel accepted and loved by another, with emotional support from the partner becoming essential, which makes it difficult to end the relationship even if there is violence, reflected in the following quotations: “When you depend on the other person, they are in the position of being able to do whatever they want with you, so they feel like whatever they do, you won't leave, nothing will happen, you'll continue to be there because you're dependent on them, so when this happens, violence is created” (Andrea. F 15-19, V); and “Compulsive ideas about affection, because I have a friend who feels the need to be with someone, but it's not because they want that person, it's just that they want affection from somewhere” (Sofia. F 10-14, V).

At the **Relational Level**, the participants indicated elements that had to do with the relationship itself that may predispose it to become a violent dating relationship or one with greater relational difficulties. These elements included cultural differences, communication issues, distrust that could lead to control of the other, or jealousy, as well as the relational experience that each partner may have. This experience would help a person to see how healthy the relationship was, as shown in the following quotations: “I think that, also supporting what they're saying, without good communication, there is no health dating relationship because there wouldn't be, if there's no communication logically, in the end, there's no good relationship. So, communication is the basis of everything, and respect” (Fernanda. F 10-14, V); and “So I think that realizing ‘it's love' would be like, I don't know, when you're in your 20s, or when you're more grown up because we have experience in relationships and with different types of people, so I think in adolescence it wouldn't be very assertive to say it would be easy to realize if it's love or not” (Constanza. F 10-14, V).

The **Family Level** was used to refer to early relational experiences to which the members of the couple may have been exposed, such as direct abuse or witnessing parental violence and experiencing the resolution of conflicts through violence as a transgenerational model, as expressed in the following: “In addition, the treatment of the family, if somebody sees domestic violence all their life, they tend to think it's normal and that leads to the idea that they'll have a relationship like that. It's worth mentioning that it occurs more than you think” (Patricio. M 15-19, I).

At the **Social Level**, the participants described being exposed to vulnerable contexts, school bullying, and mass media which showed utopian relationships that were difficult to reproduce in real life, as exemplified in the following quotation: “I find that on TV they should have to show more real relationships” (Alex. DG 10-14, I).

Finally, the participants referred to gender stereotypes; the adolescents were aware that these were still “quite rigid”, especially in the generations before them, since they recognized that social shifts have led to a more equitable and egalitarian relationship between genders, particularly in the generations contemporary with theirs. The stereotypes they mentioned were based on “machismo”, where the participants pointed out the role of “the man as provider” predominates and that of the woman is oriented to reproduction and child-rearing. In turn, they noted, with respect to sexuality, the perception that a man who has many partners is overvalued, and that this aspect is more restricted and has negative connotations for women. However, in turn, they pointed out that women perpetrate more psychological violence, like engaging in jealousy, and that men perpetrate more sexual and physical violence, and that “feminism” could also encourage violence when the feminism is extreme. One participant shared: “Speaking of the idea that generations are changing now, for example, I was raised by my father who followed the patriarchy; he put men above women, but now, as generations are changing, we are Gen Z, I have realized my machismo comments and actions and thanks to my sister, she has also supported me a lot in this issue of realizing that we are changing. There are also feminist marches now. I think that is also good because they are looking for equality between the two genders, but we must not forget that there are also radical feminists who want to be above men” (Verónica. M 10-14, I).

### 3.4 The prevention of DV

For the fourth core element, **Prevention** (31 quotations), the following categories emerged. At the **Individual Level**, the participants indicated that one way to prevent a toxic or violent relationship between two people was through knowing the other person well before starting the relationship and being aware of the behaviors that might foreshadow violence. These behaviors were called “red flags” and meant that one should not establish a relationship; red flags were exemplified in the following quotation: “I believe that there are red flags, and there are certain behaviors where people tell you how they are going to behave in the future, and you have to be aware of these warnings” (Camila. F 15-19, V).

At the **Family Level**, they considered that parents tended to sanction but not talk about violence, especially psychological violence, and that they should instill values, which was expressed in the following quotation: “There is a lack of more information for adolescents because generally, or for me at least, parents usually say ‘nobody should hit you; if they hit you, it's wrong, and you can't allow it', but they do not go deeper” (Alejandra. F 15-19, V).

At the **Structural Level**, they included school, and there appeared consensus among the participants about the need to receive information about DV through talks that begin at an early age and that were carried out in a mixed manner, including parents and questionnaires that would allow an adolescent to realize they were in a violent relationship. The school was considered a fundamental place in terms of the delivery of information, with a fundamental role in prevention, which was expressed in the following quote: “It would help if educational talks were standardized among adults and for adolescent girls together with boys because you can say, maybe they feel uncomfortable with their classmates of the opposite gender, but it is also so they realize what affects a girl and we have not shown it. Besides that, parents and adults should be included in these talks and not be isolated because they feel uncomfortable and try to avoid the topic, but rather this topic should be encouraged as something more normal to talk about” (Natalia. F 15-19, I).

At the **Social Level**, the participants alluded to the idea that helpful information should be disseminated through mass media. For example, one emphasized, “using advertising space to talk about the issue can be important” (Angel. DG 15-19, V).

### 3.5 The support networks identified by the adolescents

The fifth core element that emerged from our analysis was **Support Networks** (142 quotes) that adolescents used when faced with situations of unhealthy and/or violent relationships. The category **Friends** emerged as the main source of support for the adolescents. With friends, they would have more confidence, they would feel closer and not be judged, and there would be a feeling of loyalty and being able to count on confidentiality; however, they recognized that friends in general only advised and on many occasions could not take any more concrete action, which was exemplified in the following quotation: “I think the best psychology is friends because you can talk about things knowing that they won't tell your parents, and they also give you advice” (Antonia. F 10-14, I).

Regarding the **Family**, the adolescents claimed families were a source of support; however, they tended to turn to family in more extreme cases since they believed that turning to their parents may have the result that their parents react violently toward the aggressor or with them to end the relationship, which was expressed in the following quotation: “I think they would tell me to stay away from him. My dad would react violently; my mom wouldn't, but she would tell me to stay away” (Barbara. F 15-19, I).

Regarding **School**, the participants pointed out that education and the school system should be an important source of support; however, there appeared no consensus that this could be done concretely in all cases. They highlighted teachers as figures with fundamental capabilities, which was expressed in the following quotation: “He went to talk to his teacher about it—teachers are a fundamental source of help. He can also talk to some assistant, gardener, school inspector” (Angel. DG 15-19, V).

Then, the **Professional** category emerged. The adolescents referred to professionals in the mental health area, highlighting psychologists as accessible figures and in the second place, social workers. The participants also mentioned physicians, like psychiatrists and neurologists. The participants mentioned family health centers and community mental health centers as spaces where they could seek professional help, which was exemplified in the following quotation: “There was a psychologist at the Family Health Center, and you can talk to her in these cases” (Carla. M 15-19, I).

As for **Institutions**, they stated that there were different institutions that could protect a person who was experiencing violence in their relationships, among them police officers, the investigative police, health centers, family courts, the National Youth Institute (INJUV; i.e., the government agency responsible for designing and implementing public policies related to youth), and the Ministry of Women and Gender Equality. They claimed that the laws protected women more than men, and they knew of different ways to file complaints (e.g., phone numbers, keywords), which was exemplified in the following quotation: “The Ministry of Women was also created for a reason, to highlight a type of violence against women and to seek the equality that men have historically had, in my opinion” (Carolina. M 15-19, V).

Some adolescents stated they were **Without Support Networks**, and in the case of DV, they would not use the above networks because of a distrust in institutions, fear of reprisals mainly from the family, and shame in recounting what they were going through. A participant shared: “Out of fear of the reprisals that could be behind it [I would stay quiet], because one does not know the type of family one has, there is also no support in the establishments, especially in the public ones” (Consuelo. F 15-19, I).

### 3.6 “What should be done” in the face of DV, according to the adolescents

Finally, the fifth core element that emerged was **What Should Be Done About Dating Violence**. In one category that emerged, “As a Witness to Violence”, the participants shared that it was difficult to help a person who was suffering violence, but, in general, there was a consensus that such a relationship could not be maintained. They said that in the case of psychological violence, the person should seek help at the family and professional levels, and some even mentioned couples therapy as an option. In terms of physical violence, they emphasized that this should always be reported, and the relationship should be terminated, which was exemplified in the following quotation: “It's hard to help someone who doesn't know what might happen to them. Advising them and telling them that's wrong and ‘you have to do something about it', you have to try to reconsider what's happening to you … but I think that would help them. Try to confront the violence, confront the person, and tell them you're hurting me, try to make them realize what they're doing” (Soledad. M 10-14, I).

Finally, in this same core, the category “As a Victim of Violence” emerged, which highlighted that it was important to “love oneself first” in order not to accept violent behavior in a dating relationship. The participants separated psychological and physical violence and placed the latter at a higher level of severity, stating that at a lower level one should listen to the other and improve communication and then seek help at the family and professional levels and, in cases of greater severity, end the relationship and file a complaint. One person shared: “I think that depends on the person and the degree of violence because if the violence is still minor, you can talk things over as long as you are willing to communicate; otherwise, the best thing to do is to end the relationship” (Kate. DG 15-19, V).

## 4 Discussion

Despite their youth, the participants in this study demonstrated a command of the study topic and could characterize and distinguish between healthy and unhealthy relationships, perhaps because of the knowledge imparted by schools or the mass media in recent decades or because of their own and their peers' early relationship experiences, coinciding with the theoretical framework (Eaton et al., 2010; Ministerio de Desarrollo Social, 2015). Furthermore, no comparison was made here between age groups and gender since no differences were found between the comments of the younger and older adolescents, or between those of the boys, girls, and different groups. This is a valuable finding since it accounts for an intersubjective perception of these phenomena, which was shared.

However, probably because research and prevention programs have focused mainly on DV rather than on the promotion of HDRs (Eaton et al., 2010), it appeared easier for the participants to express their experiences and opinions about violent relationships than about healthy relationships; when describing a healthy relationship, they tended to do so in terms of an unhealthy relationship, as other authors have observed (Finkel et al., 2009; Noonan and Charles, 2009). This demonstrates the need to improve policies for wellbeing within the culture, with knowledge about wellbeing, health, and positive relationships being promoted. It is also important to avoid leaving gaps in this regard by defining wellbeing as “the absence of …”. This was the case with health, which for many years was defined as the “absence of disease” rather than as a concept that evolves as a result of certain determinants, and that can be understood as a process that in turn constitutes a resource in people's life development (Herrero, 2016).

### 4.1 Conceptualization

Nevertheless, the adolescents posited that an HDR is a process in which affection, love, and care for the other are present. They also suggested that an HDR implies personal development and independence of one member of the couple from the other in a way that favors self-knowledge and wellbeing. They recognized that an unhealthy relationship implies behaviors contrary to healthy ones, and an unhealthy relationship was associated with violent behavior. This recognition would be independent of the sociocultural context since many types and manifestations of DV are shared by most Latin American cultures (Jaskulska et al., 2022; Tobar Lasso et al., 2022).

We found it striking that the participants emphasized that not everything in relationships is polarized; some participants described how, in a relationship, it was expected that there would be nuances and that conflicts and discussions would arise, making explicit an integrative perception of the couple's relationship, in accordance with postmodern systemic literature, which evidently draws the attention of such young people (Levant and Silverstein, 2001). They made it clear that they believed that developing nonviolent conflict resolution skills and incorporating these subtleties into relationships was more vital than idealizing or succumbing to romantic relationship myths, essentially agreeing with past authors (Sanhueza, 2016).

### 4.2 Protective factors

The adolescents claimed that they had internalized protective factors but found it easier to describe risk factors, consistent with what was previously pointed out regarding the lower cultural knowledge about people's wellbeing and how to promote it. Regarding the former, they pointed to emotional independence and self-love as central aspects; this was understood as the ability to maintain adequate self-esteem regardless of the acceptance of others, and the ability to set limits while safeguarding personal integrity—issues also raised by other authors (Dardis et al., 2014; Hébert et al., 2017; Malhotra et al., 2014; Spencer et al., 2019; Taylor et al., 2017; Vagi et al., 2013). The participants also mentioned “affective responsibility” as an important relationship protective factor; they understood this as caring for the other, not causing harm, and nurturing trust (Gila-Ordóñez and Callejón-Chinchilla, 2018; Hernández-González, 2012).

The adolescents stated that today's society is active, and that a cultural shift is occurring that fosters respect and rejects violence. This had enabled them to become informed about which behaviors are and are not beneficial in a dating relationship, and to begin empowering themselves in this respect.

### 4.3 Risk factors

Regarding risk factors, the participants appeared aware that if they experienced violence in the family, witnessed violence between parents, or had an upbringing based on abuse, they would tend to normalize relationships in which violent behavior was a valid tool for resolving conflicts. They stated that they were aware that these models are inadequate but felt that they were still prone to repeat them, which was considered a serious risk factor due to the early internalization of these behaviors; this commentary appeared consistent with what has been described as a risk factor in the literature (e.g., Sianko et al., 2019). One positive aspect of this was that, by indicating their awareness about this issue, they appeared to be one step ahead with respect to wanting to repair their own histories in order to forge a different path, an idea reinforced by past authors (Espinoza et al., 2019; Froidevaux et al., 2020; Katz et al., 2017; Zamudio, 2012; Muñoz-Rivas et al., 2019). However, not all the responsibility was reported to fall on the family. The adolescents argued that anyone could express or be exposed to violence, but that this would be intensified in adolescence because of the characteristics inherent in this stage, added to the idealization of romantic love and dating relationships by the mass media, which coincides with the theory (Flores, 2019; García et al., 2019; Villarejo-Carballido et al., 2022).

Two additional factors merit particular analysis: The first is emotional dependence. The participants stated that, when faced with the fear of not feeling accepted or the pain of not feeling loved, they would be willing to accept being in a violent relationship (Pérez-Pimienta and García y Barragán, 2020). The second factor was jealousy—a complex construct associated with the irrational fear of losing one's partner—which generates distrust, insecurity, impairment, unpleasant emotions of distress, and controlling behaviors over the object of one's desire, among other things. Jealousy, which is typical of but not exclusive to the adolescent stage, greatly hinders couple relationships and is an important substratum of violence. Jealousy, studied by many, has been associated in other samples with an increased risk of self-harm and suicidality in adolescence (Brar et al., 2022; Baker et al., 2014; Garrido-Antón et al., 2020; Hernando-Gómez et al., 2016; Zulic-Agramunt et al., 2022).

Another interesting aspect mentioned by the adolescents was that a low capacity for emotion regulation favored impulsiveness and the expression of violent behavior. Attempts have recently been made to reverse this concern, using models such as respectful upbringing and the recognition and management of emotions in boys and girls, consistent with the literature in which impulsiveness and empathy have been highlighted as determining factors in the wellbeing of relationships (Dodaj et al., 2020; Glowacz and Courtain, 2020). In this respect, the adolescents mentioned prevailing gender stereotypes in Chilean society associated with machismo and the patriarchal culture in which the country is immersed (Calvin et al., 2013). Although they recognized that there are discourses, proposals, and actions that promote equitable relations, there remains, in practice, in the generations before them, rigidity in gender roles based on machismo, which made it very difficult to restructure beliefs about how men and women should be. The adolescents had been raised under these models, but with new social proposals, they took a critical stance on traditional couple relationships and appeared committed to change and respect gender diversity. Referring to the latter, they emphasized that gender diverse people can relate in a similar way to heterosexual couples, and they were aware of the stigma that fell on minorities and how this could affect relationships and people, according to the literature (Claussen et al., 2022; Dardis et al., 2014; Fernández-Antelo et al., 2020; Haglund et al., 2018; Taylor et al., 2017).

We also note that the adolescents recognized that violent behaviors were supported by the normalization they received in some social contexts of greater psychosocial vulnerability, and fewer economic resources were mentioned by the participants as being among the risk factors at the social level. Essentially, unmet basic needs lead to stress (Claussen et al., 2022; Taquette and Monteiro, 2019) and victimization (Paat and Markham, 2016). Regarding violent behaviors such as sexual assault, they were not mentioned in the discussion nor were they explored in depth, as the focus was on healthy relationships, and the topic is beyond the scope of this study.

### 4.4 Prevention factors

Regarding the prevention of violence, the adolescents (i.e., mostly the girls), were attentive to the signs that threaten healthy relationships, described in the prevention core at the individual level as “red flags” (Guerrero et al., 2021). The participants claimed it was important to know one's partner's attitudes and values to avoid continuing in the relationship if there was a possibility of violence; these attitudes and values would be detected through the red flags. This finding is encouraging and satisfactory, especially considering that the participants were young adolescents and that some effective prevention campaigns use the socialization of such alarms to instruct young people regarding violence prevention and detection (Carlyle et al., 2020). However, this ability to give up a relationship was directly related to individual characteristics, especially emotional dependence, as reported by the adolescents, and this is consistent with other studies which showed that emotional dependence is directly related to DV and inversely related to life satisfaction (Marcos et al., 2020; Ponce-Díaz et al., 2019).

### 4.5 Education on prevention

Concerning the role of the educational system in prevention, the participants considered that school was a significant space for conveying information and for personal development, which should begin at an early age, as proposed by other studies (Gila-Ordóñez and Callejón-Chinchilla, 2018), suggesting that school employees play a relevant support role in preventing DV.

It is helpful to build and to use support from peers and teachers, not only to provide knowledge about violence and social skills, but also to enhance school climate and relationships that can become direct sources of support, highlighting the importance not only of cognitive aspects, but also of emotional aspects and interpersonal relationships within the school (Jankowiak et al., 2020).

One of the oldest prevention programs—“Skills for Violence-Free Relationships”—was developed in the United States by Levy in 1984. It is aimed at students from the seventh to the 12th grade and has a gender perspective that presents boys as the main perpetrators of violence and girls as the main victims (Martínez and Rey, 2014). Significantly, the adolescents in the present study claimed this polarity is less evident now, stating that some women also commit acts of violence but do so in different ways than men, making men's suffering more invisible and giving less attention to female-perpetrated violence. They also emphasized that gender-diverse couples may experience violent relationships, clarifying an inclusive and integrative perspective that encompasses a range of possibilities at the relational level.

While numerous intervention programs now exist, evaluations in high-income countries are generally more likely than those in low- and middle-income countries to assess programs' effects on victimization and perpetration of adolescent DV (i.e., rather than on victimization alone). They are also more likely to include both boys and girls, rather than being single-sex programs, and while the evaluations have reported a significant preventive effect on at least one outcome for adolescent DV, the findings in the literature generally suggest that more research is needed to shed light on how these programs work and to identify whether the effects can be generalized across different settings, outcomes, and subgroups (McNaughton et al., 2020; Russell et al., 2021; Solomon et al., 2021). It should be noted that DV prevention programs are facilitated in Chile; however, these generally target the university population and occur late for early prevention (Lara and Providell, 2020; Vizcarra et al., 2013). The family usually performs a protective or a risk function, according to family functionality, in terms of prevention (Gila-Ordóñez and Callejón-Chinchilla, 2018). Our adolescents stated that the education provided in schools should include families since, instead of instilling values, they tended to, in many cases, punish the young people by blocking the possibility of talking about the issue (Bolívar et al., 2017). At the social level, the participants considered that the media should promote the questioning of violence, putting the issue on the table and being more careful with gender stereotypes and machismo, which were considered risk factors in previous studies (Cruz-López and García-Meraz, 2022; Cuadrado-Gordillo and Martín-Mora-Parra, 2022). In this regard, our participants highlighted that cultural differences in a couple could influence their ways of relating; this is striking, since Chile, in addition to having a historical presence of indigenous peoples, is currently a country with a significant migrant population, where the phenomena of acculturation and encounter between different cultures is observed in various regions of the country (Sanhueza et al., 2022). Although the cultures are mostly Latin, they have different worldviews regarding machismo, patriarchy, and gender roles, which increase the complexity of how to address relational phenomena (Ghafournia, 2014).

### 4.6 Support networks

The adolescents claimed to turn to support networks depending on the perceived level of severity of the violence, considering physical violence to be more serious than psychological violence. In this regard, they mentioned that the most reliable source of support was friends, although, in general, friends could not solve the problem. Friends were highly valued as they listened to one without judgment and did not pressure one to break up (as the family would), which is in accordance with the literature (Rodríguez et al., 2018). This also invites us to ask ourselves, “What is happening with families and the bond with adolescents?” Although it would be expected that, because of their developmental stage, adolescents would consider their friends first, it would be expected that the family, because of proximity, would be the first network to which they would turn; however, many appeared afraid of the reprisals they might receive from their family. This led to the idea, again, of the importance of including families in intervention programs, and the relevance of the bond with parents and of fostering and maintaining a truly secure attachment with them throughout life so that they may be considered the main support network in times of adversity (Doucette et al., 2021).

The adolescents also cited the public mental health centers in the Chilean health system as a support network and considered professional psychologists accessible figures to whom teachers could turn to in case they need help. For the adolescents, teachers—because of proximity—should be support networks as much as, or more than, health professionals (Jankowiak et al., 2020), but apparently teachers would not act effectively as a source of help. However, as previously noted, teachers are the focus and hope for future interventions in the area of prevention.

The adolescents also shared about institutions that work with intimate partner violence, such as the police or INJUV, among others; they knew that there was no law against intimate partner violence but that they could still file a complaint if there was mistreatment, which has also been raised in other studies carried out in Chile (Valdivia-Peralta et al., 2018), to ensure that institutions and professionals dealing with DV are proficient in all areas of intervention (promotion, prevention, treatment, rehabilitation). DV must remain visible at the public policy level. Additionally, it appears crucial to break with the health model that prioritizes repairing harm over promoting wellbeing. This model is evident in the allocation of resources at the state level, at least in Chile, which neglects in-vestment in community-based policies that support wellbeing and healthy relationships, as recommended by certain lifelong prevention programs (Schubert, 2015).

Note that although most of the participants knew that support networks existed and knew what they must do if they were witnesses to or victims of violence, some adolescents did not trust their networks and instead dealt with DV alone, or not at all (Rodríguez et al., 2018). This is concerning, given that social support has been identified as playing a fundamental role in the wellbeing of victims, and is even known as a moderator of individuals' psychological wellbeing (Garcia et al., 2014; Pariartha et al., 2022).

Finally, many of the participants valued the space they were given in the focus group. Just having a conversation on the subject was seen as beneficial because it highlighted the importance of healthy relationships and discouraged the normalization of violent behavior in relationships.

### 4.7 Limitations

The composition of our focus groups in terms of gender stratification can be considered a study limitation, as it could have reduced the possibility of wider dialogue and discussion. Nonetheless, since the groups were randomly stratified, it was deemed more important to proceed in this manner to lower the likelihood of putting possible aggressors and victims in the same space, or couples in the same group. Regarding the group with gender diversity, it was thought that they would be more protected in a group that only contained adolescents of diversity since this would reduce the risk of exposing them to unknown adolescents who could make discriminatory comments, considering that, in Chilean culture, there is still conservative thinking in some families. Selection bias may have resulted from this, as parents and adolescents with greater knowledge of the subject may have been more inclined to participate because they were aware of the phenomenon and significance of DV. This could account for the adolescents' well-reasoned responses, which were mostly in line with the literature and disassociated from certain cultural myths. Finally, there could also have been limited access to adolescents who had been exposed to high levels of violence or even abuse, given the fear of making the problem visible or the pressure to denounce and expose the problem publicly.

## 5 Conclusions

This study has highlighted the difficulty of describing and conceptualizing the meaning of HDRs, given that the adolescents in this research provided more information about violent relationships than about healthy relationships. This illustrates the need for ongoing research to create an HDR construct that can be promoted in different cultures.

Listening to the adolescents allowed us to demonstrate their capacity for critical analysis and level of empowerment concerning dating relationships and what they considered to be healthy or harmful in such relationships. This “opens the door” to implementing measures focused on prevention and promotion adapted to adolescents, which allow “laying solid foundations” from the beginning of early relationships, which can translate into healthy family relationships in the future and prevent gender violence, child abuse, complex traumas, and suicidality, among other outcomes. In practice, relationships in adolescence are the first step to building a future couple and family in adulthood and a healthy protective relational network for future generations.

This study also made it possible to gather information on what adolescents expect from an HDR and to meet their educational needs, highlighting the need to promote new ways of conceiving couple relationships from an ecological model at cultural, social, institutional, family, and individual levels, and with a gender perspective, too, to foster positive experiences equitably and inclusively. Overall, it appeared clear that the adolescents were not mere recipients of information; they articulated needs for commitment and support in their developmental processes and establishing a couple relationship was a central subject that could determine their wellbeing or cause problems, particularly in their mental health and future relationships. This highlights the importance of considering adolescents' opinions and experiences when formulating public policies. These policies should prioritize the implementation of programs that promote HDR and strengthen positive behaviors while also focusing on preventing violence. Rather than focusing solely on what should be avoided, a culture that fosters wellbeing and recognizes relationships as the fundamental axis in maintaining people's health and happiness might be increasingly co-created.

## Data Availability

The raw data supporting the conclusions of this article will be made available by the authors, without undue reservation.
